# The Regulation of the Albomycin and Desferrioxamine E Biosynthesis in *Streptomyces globisporus* bja209

**DOI:** 10.3390/molecules30193871

**Published:** 2025-09-24

**Authors:** Julia A. Buyuklyan, Mikhail V. Biryukov, Yulia V. Zakalyukina, Artemy A. Sacharov

**Affiliations:** 1 Department of Biotechnology, Sirius University of Science and Technology, Olympic Ave., 1, 354340 Sirius, Russia; buyuklyan20@gmail.com (J.A.B.);; 2 Department of Biology, Lomonosov Moscow State University, Leninskie Gory 1, 119991 Moscow, Russia; 3 Department of Soil Science, Lomonosov Moscow State University, Leninskie Gory 1, 119991 Moscow, Russia

**Keywords:** albomycin δ2, deferoxamine E, C-1027, biosynthetic gene cluster (BGC), *Streptomyces globisporus*, linear plasmid

## Abstract

We identified *Streptomyces globisporus* bja209 through a targeted screen of actinomycetes from natural habitats using an *E. coli* JW5503 ΔtolC DualRep2(c) reporter strain. This strain produced antibacterial compounds whose action depended on the growth medium. HPLC-MS and genomic analysis revealed two metabolites: albomycin δ2 (a translation inhibitor) and desferrioxamine E. The latter induced the SOS response. Desferrioxamine E exhibited a narrow spectrum of antagonistic activity against carbapenem-resistant *A. baumannii* and *C. michiganensis*, and its production was critically regulated by iron concentration. Notably, the structurally similar desferrioxamine B was inactive. Contrary to previous reports, pangenome analysis of published GenBank genomes revealed that albomycin BGC is restricted to specific *S. globisporus* strains and not present in other Streptomycetes phylogenetic clades. The C-1027 BGC was found in a large linear plasmid (165.5 kb) of the *S. globisporus* bja209 strain and also found exclusively on linear plasmids in some of the published *S. globisporus* genomes.

## 1. Introduction

Since the mid-20th century, using the so-called “Waksman platform” has led to the identification of hundreds of promising molecules. However, over time, the rate of discovering novel compounds using conventional approaches has significantly declined, with researchers increasingly encountering already known substances. This led to the perception that such screening methods had been exhausted. Meanwhile, the pharmaceutical industry believed it possessed a sufficient range of compounds to meet any needs in antimicrobial therapy [1,2,3].

The spread of multidrug resistance among pathogenic microorganisms to clinically approved antibiotics has necessitated the search for novel compounds with distinct targets and mechanisms of action to overcome prevalent resistance mechanisms. To address this, highly sensitive screening systems have been implemented, enabling the determination of antibiotic mechanisms of action at the early screening stage, thereby identifying the most promising molecules for further development. One promising approach is target-based high-throughput screening, which focuses on discovering antibacterial agents that disrupt key processes in bacterial genetic information flow, such as replication, transcription, and translation. Currently, the pDualrep2(с) reporter system has proven effective, detecting compounds that induce the SOS-response or inhibit protein biosynthesis through the expression of fluorescent protein genes [4].

Such methods not only facilitate the discovery of novel antimicrobial metabolites but also help to unveil the potential of already known molecules, which, in turn, may expand their clinical applications.

Soil bacteria of the phylum *Actinomycetota*, particularly the genus *Streptomyces*, represent a rich source for novel antibacterial discovery due to their capacity to produce a plethora of secondary metabolites. Consequently, they are the source of approximately 75% of clinically utilized antibiotics [5,6,7]. Actinomycetes are Gram-positive, soil-dwelling microorganisms characterized by an exceptionally high genomic GC content (approximately 70%) [8,9]. These organisms possess notably large genomes (5–10 megabase pairs), with a substantial proportion of their coding capacity devoted to secondary metabolite biosynthesis gene clusters [10,11,12].

The advent of genetic engineering technologies and high-throughput genomic sequencing has revolutionized antibiotic discovery, enabling not only the identification of novel compounds but also systematic investigation of biosynthetic gene clusters and their associated regulatory elements. A particularly innovative approach involves the activation of “silent” antibiotic clusters through exposure to signaling molecules, including metal ions, subinhibitory antibiotic concentrations, and specific culture medium components [13].

In the present study, we identified the strain *S. globisporus* bja209 capable of biosynthesizing two compounds with distinct mechanisms of action—albomycin δ2 and desferrioxamine E. The production of each metabolite was regulated by specific culture medium components. Furthermore, using a reporter system, we have for the first time characterized desferrioxamine E’s mechanism of action, which was associated with induction of the SOS-response in *E. coli*. Additionally, our findings demonstrated that both desferrioxamine E and albomycin δ2 exhibit efficacy against a range of clinically relevant bacterial pathogens.

## 2. Results

Initial screening of bja209 against the *E. coli* JW5503 ΔtolC DualRep2(c) reporter strain revealed that, depending on the cultivation medium, it produced metabolites with different mechanisms of antagonistic activity. Cultivation in ISP3 medium yielded a compound inducing SOS-response activation, indicating production of DNA replication-interfering metabolites, while ISP6 medium cultivation produced metabolites demonstrating translation inhibition activity. This finding highlights several key aspects: media-dependent regulation of specialized metabolite biosynthesis and the strain’s genetic capacity for producing mechanistically distinct bioactive compounds. The results offer new perspectives on how actinobacteria physiologically adapt, how biosynthetic gene clusters are evolutionarily conserved, and how environmental cues activate silent metabolic pathways.

### 2.1. Genome Features and Phylogenomic Analysis of S. globisporus bja209

The genome of strain bja209 comprises a 7,865,380 bp chromosome and a 165,749 bp linear plasmid, totaling 8,031,129 bp. The DNA G+C content is 71.42%, which is consistent with the G+C content of the genus members of *Streptomyces* [14]. The chromosome includes 6 ribosomal operons, 83 tRNA, 1 tmRNA, and 7114 coding sequences.

Neighbor-joining phylogenetic analysis based on both 16S rRNA and whole-genome sequences from the TYGS database and GeneBank confirmed that bja209 belongs to the species *S. globisporus*. Accordingly, we designate this strain as *S. globisporus* bja209.

**Figure 1:**
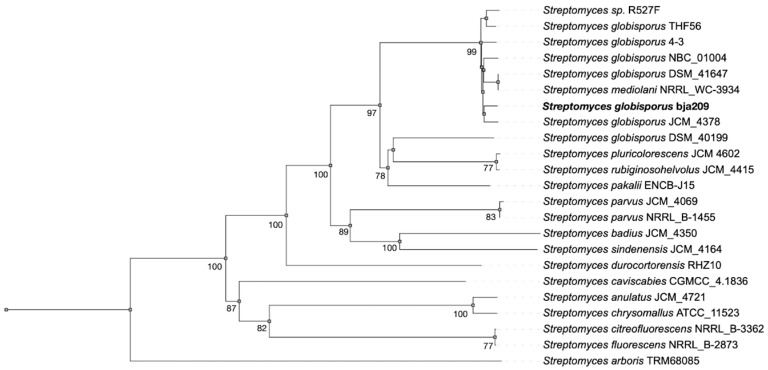
Phylogenetic tree based on whole-genome sequences from bja209 within the evolutionary radiation of type strains and closely related Streptomyces spp. (bootstrap support > 60% at all key nodes).

OrthoANIu analysis revealed 99.49% and 99.48% average nucleotide identity (ANI) between bja209 and the type strains *S. globisporus* NBC_01004 and *S. globisporus* THF56, respectively (Table S1). These values significantly exceed the established 95% ANI threshold for species delineation, further supporting its taxonomic assignment (Figure 1) [15].

*S. globisporus* bja209 and its phylogenetically related neighbors—*S. globisporus* NBC_01004 and *S. globisporus* THF56—harbor large linear plasmids [16,17]. Linear plasmids localize to chromosomal terminal regions and possess autonomous replication origins (ori) alongside dedicated replication machinery, including sequence-specific DNA-binding proteins [18]. The plasmid can exist as an autonomous extrachromosomal element capable of self-replication and conjugative gene transfer or, alternatively, integrate into the host chromosome as an associated episome [19].

**Figure 2:**
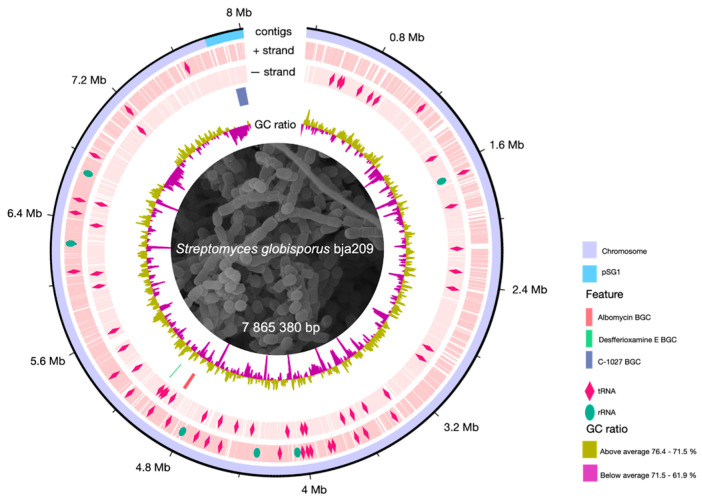
Circular map of the *S. globisporus* bja209 chromosome. Outer ring: genomic coordinates (chromosome and linear plasmid pSG1). Middle rings: locations and identities of tRNA (pink rhombus) and rRNA codons (green oval) and key biosynthetic gene clusters (BGCs) discussed in this study (albomycin, desferrioxamine E, C-1027). Inner ring: GC skew.

Annotation of secondary metabolite BGCs through antiSMASH 8.0.1 [20] revealed 33 putative BGCs, four of which showed high probability (bootstrap support 85–95%) of encoding compounds with demonstrated antimicrobial activity: albomycin δ2, desferrioxamine E, griseobactin, and C-1027 antibiotic. The remaining BGCs were predicted to encode pigments, osmoprotectors, and incomplete clusters (30–60% core biosynthetic genes) (Figure 2). These computationally prioritized antimicrobial BGCs served as primary targets for experimental validation of strain-derived metabolites.

### 2.2. Phenotypic Characterization of S. globisporus bja209

**Figure 3:**
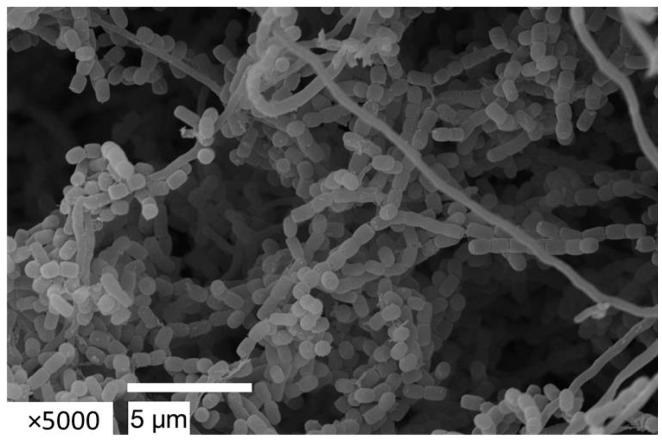
Spore chain of *S. globisporus* bja209 observed by scanning electron microscopy (SEM). After 14 days of incubation on ISP3 medium at 28 °C.

The strain *S. globisporus* bja209 is an aerobic, Gram-positive, non-motile actinobacterium that exhibits robust growth on ISP3 and ISP6 media, moderate growth on ISP5, and forms abundant substrate mycelium (white to beige) and well-developed aerial mycelium (beige) within 2–3 days of cultivation (for detailed cultural characteristics, see Table S2). Scanning electron microscopy confirmed the formation of spore chains typical of the genus *Streptomyces* (Figure 3).

Here, we investigated the regulatory mechanisms governing secondary metabolism, which is a more complicated version of primary metabolism and has similar regulation. As an accessible example for primary metabolic activity, we analyzed carbohydrate utilization profiles using the disc-diffusion method (Table S3), comparing strain bja209 with the previously characterized albomycin-producing strain *S. globisporus* 4-3 from our laboratory. A key metabolic distinction was observed: unlike strain 4-3, bja209 demonstrated lactose utilization capability (a trait not yet documented in type strains of this species).

Comparative genomics of *S. globisporus* strains bja209 and 4-3 revealed the genetic basis for their metabolic differences. While both strains possess a similar suite of β-galactosidase genes, a critical mutation was identified in strain 4-3: its *lacZ3* gene is N-terminally truncated, disrupting the enzyme’s tetrameric assembly and probably explaining its inability to utilize lactose (Figure S1) [21,22]. This mutation appears to be rare among *S. globisporus* strains, found only in strains 4-3 and THF56 (no data on lactose metabolism is available for THF56). There is data on lactose metabolism available only for *S. griseus* DSM40395, and deletion in the *lacZ3* gene probably leads to a lactose-negative phenotype [23].

Further metabolic profiling revealed that bja209 also has a reduced capacity to utilize galactose and arabinose compared to strain 4-3. For galactose, this is likely due to a difference in gene dosage, as strain 4-3 possesses two copies of the *galactokinase* gene versus a single copy in bja209 [24]. For arabinose, the phenotypic difference is not due to enzyme sequence but rather to potential differential expression or DNA-binding strength of the transcriptional repressor AraR, which probably more strongly suppresses the catabolic pathway in bja209 (Figure S1). This highlights how regulatory mechanisms, beyond mere gene presence, drive metabolic heterogeneity.

The study provides a framework for linking genomic plasticity to phenotypic diversity in soil bacteria, with implications for understanding niche adaptation and engineering metabolic pathways in biotechnological applications.

### 2.3. Antibiotic resistance profile of S. globisporus bja209

The antimicrobial resistance profiles of individual strains fundamentally shape the defined microbial composition and ecological niche occupation. Crucially, antibiotic-producing microorganisms must carry self-resistance mechanisms to avoid self-inhibiting during bioactive metabolite production. These adaptive strategies include target site modification, efflux pump overexpression, and enzymatic inactivation of the antibiotic. This intrinsic link between production capability and self-resistance makes antibiotic susceptibility profiling a useful tool for identifying and characterizing antibiotic producers. Using standardized disc-diffusion assays, we evaluated the susceptibility of strain bja209 to a panel of clinically relevant antibiotics representing major drug classes (Table 1).

The antibiotic resistance patterns of the bja209 strain reveal a sophisticated interplay of enzymatic inactivation and target-specific adaptations, reflecting evolutionary pressures in competitive soil environments. Most notably, the strain demonstrates broad resistance to β-lactam antibiotics, including oxacillin and carbapenems, which is effectively reversed by clavulanic acid co-administration. This phenotype is mediated by three genomic β-lactamases: a chromosomally encoded Class C enzyme with broad-spectrum activity against penicillins and cephalosporins, and two terminal Class A β-lactamase genes, one of which requires post-translational activation. The clavulanate-sensitive inhibition profile confirms that β-lactam degradation, rather than target modification or membrane impermeability, drives this resistance.

Contrasting with its β-lactam resistance, bja209 shows susceptibility to most aminoglycosides while maintaining specific resistance to streptomycin and streptothricin. Genomic analysis revealed the absence of specific ribosomal mutations (intact *rpsL* gene) and instead identified an aminoglycoside aminotransferase, responsible for streptomycin inactivation. The streptothricin resistance is conferred by a dedicated streptothricin acetyltransferase, demonstrating exceptional substrate specificity without cross-resistance to other aminoglycosides. Additionally, the strain carries two variants of sulfonamide-resistant dihydropteroate synthases (*sul1* and *sul2*), explaining its tolerance to synthetic antimicrobials.

The strain exhibits variable susceptibility to fluoroquinolones, demonstrating notable resistance to norfloxacin despite these antibiotics targeting universal bacterial enzymes (DNA gyrase and topoisomerase IV) that typically induce SOS responses—consistent with our initial reporter assays. This species-specific resistance pattern arises from characteristic mutations in DNA gyrase and topoisomerase IV.

The collective resistance profile of bja209, which spans β-lactams, aminoglycosides, sulfonamides, and fluoroquinolones, is primarily mediated through enzymatic inactivation.

### 2.4. Identification of Active Compound in S. globisporus bja209

**Figure 4:**
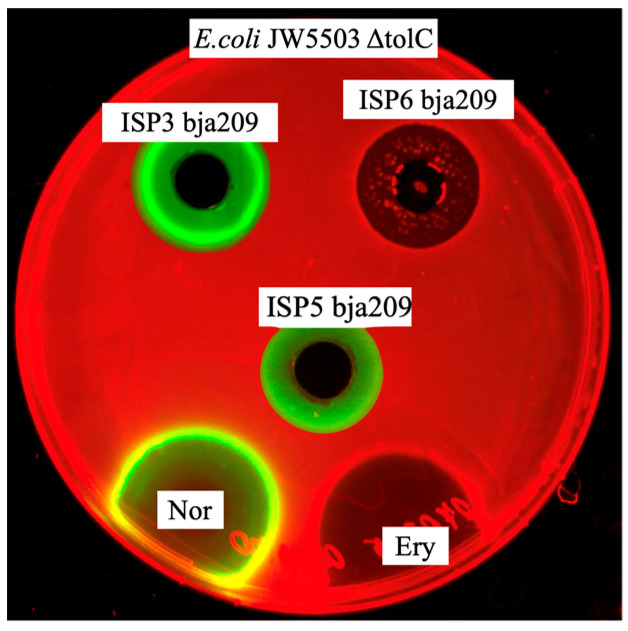
Activity of crude broth of bja209 against *E. coli* JW5503 ΔtolC pDualrep2. Extract from ISP3/ISP5 cultivation medium induced the SOS response, and ISP6 cultivation medium induced Katushka2S signal in the test strain. The agar plates were spotted with erythromycin, 5 μg (Ery), and norfloxacin, 1 μg (Nor). Katushka2S and TurboRFP signals visualized via ChemiDoc MP with red and green colors, respectively.

Initial screening of bja209 revealed distinct antagonistic activity patterns against the *E. coli* JW5503 ΔtolC test strain when cultivated on different ISP media formulations. The mutant’s impaired efflux system enhances its sensitivity to intracellular-acting compounds, facilitating the detection of novel bioactive metabolites. Metabolites produced by bja209 exhibit distinct mechanistic action dependent on cultivation media. Extract from ISP3/ISP5 cultivation medium induced the SOS response, and ISP6 cultivation medium induced Katushka2S in the test strain (Figure 4).

HPLC-HRMS analysis of the culture filtrate exhibiting translation-inhibiting activity revealed the presence of albomycin δ2, while the SOS response-inducing fraction yielded the DNA-damaging compound desferrioxamine E and C-1027. This result corresponds with genomic data of BGC.

Crude extract was purified through SPE and HPLC with the following HRMS analysis for the active HPLC fraction. The separation was achieved using reverse-phase HPLC on a Luna C18(2) column with a water–acetonitrile gradient system. The elution program consisted of 5–10% B (0–2 min), 10–15% B (2–22 min), 15–95% B (22–26 min), and isocratic elution at 95% B (26–28 min). Detection was performed at 290 nm, with albomycin δ2 eluting at 11.320 min. HRMS analysis of the translation-inhibiting fraction identified a metabolite exhibiting positive-ion mode adducts [M + H]^+^ and [M + 2H]+ at 1046.309095 m/z and 523.659953 m/z, corresponding to a monoisotopic molecular mass of 1045.3047 Da. The specific absorption spectrum at 278 nm and searches in the chemical databases (NPAtlas, Dictionary of Natural Products, and PubChem) allowed us to determine the molecular formula C_37_H_52_FeN_12_O_18_S that corresponds to albomycin δ2 (for detailed HPLC fractionation, see Table S4).

The SOS-responsive compound from ISP3-grown cultures was purified by HPLC on a C18 column using a water (A)/acetonitrile (B) gradient as follows: 5–20% B (3 min), 20–60% B (6 min), 60–95% B (3 min), hold at 95% B (4 min). Deferoxamine E eluted at 14.1 min. The detected mass did not match the expected profile for C-1027 (844.27 Da) but fully matched the profile for desferrioxamine E. HRMS analysis revealed ion adducts [M + H]^+^ and [M-H]^−^ at 601.259708 *m*/*z* and 599.346364 m/z and was clearly distinct from the mass of desferrioxamine B (561.3715 *m*/*z*). Coupled with the characteristic UV absorption at 272 nm, database searches (NPAtlas, Dictionary of Natural Products, and PubChem) allowed us to determine the molecular formula C_27_H_48_N_6_O_9_ that corresponds to desferrioxamine E (for detailed HPLC fractionation, see Figure S3).

An additional assay was performed to determine the expected desferrioxamine E chelating activity. The active fraction demonstrated a reddish halo on a solid chromazurol medium that confirmed the molecules’ ability to chelate Fe from the Fe–chromazurol complex (Figure S5).

Thus, in addition to the DNA-targeting compound C-1027, which induces the SOS response via direct DNA damage, the strain unexpectedly produced an SOS-activating compound, which was identified as desferrioxamine E—an iron-chelating siderophore.

There are reports about desferrioxamine E antagonistic activity against *M. smegmatis* [26], but the mechanism of action has not yet been investigated. In our research, desferrioxamine E confirmed activity against *M. smegmatis*, clinically relevant isolates of *A. baumannii* blaNDM, and the phytopathogenic bacterial strain *C. michiganensis* (Figure S1). Other bacterial strains were not susceptible to desferrioxamine E. This result indicated a narrow spectrum of activity for desferrioxamine E, which may explain the limited prior research on its antibacterial properties.

Desferrioxamines’ BGC is widespread among of streptomyces genomes. Earlier, we had already investigated the strain *S. globisporus* 4-3 with the same BGC but without any SOS response activity. HRMS analysis of 4-3 crude extract revealed only desferrioxamine B. These findings indicate that the ability to induce the SOS response is specific to the E form of desferrioxamine and is not exhibited by the B form. Currently, there is no information available about desferrioxamine E’s peculiar mechanisms of action.

Although the precise cellular targets of desferrioxamine E have not been fully characterized, our studies provide mechanistic insights through comparative screening against antibiotic-resistant strains. The observed SOS induction appears unlikely to involve DNA intercalation, given the steric constraints of the desferrioxamine E molecule. Activity profiling against quinolone-resistant (nalidixic acid-resistant) and novobiocin-resistant *E. coli* strains indicated that desferrioxamine E maintains antagonistic activity against quinolone-resistant variants but shows reduced efficacy against novobiocin-resistant strains. This resistance pattern suggests that desferrioxamine E may primarily target DNA gyrase (topoisomerase II), potentially inducing replication stress and subsequent SOS activation (Figure S5). These findings may represent a previously unrecognized mechanism of siderophore-mediated antimicrobial activity, which extends beyond iron chelation to include direct interference with essential DNA topology maintenance systems. The conclusions drawn, while suggestive of a potential mechanism of action for desferrioxamine E, remain speculative and must be verified through targeted biochemical assays, including enzyme inhibition, super-coiling, and cleavage assays, resistant clones, and SNP analysis. They were not carried out in the current study but will be the focus of future investigations.

### 2.5. Regulation Cues Inducting Albomycin δ2 and Desferrioxamine Biosynthesis

As previously observed, differential reporter induction patterns—indicating distinct antibiotic production—emerged when *S. globisporus* bja209 was cultured on ISP5 versus ISP6 media. To identify the key regulatory factor governing this metabolic switch, we designed a synthetic media experiment by systematically omitting individual components: soluble starch, KNO_3_, MgSO_4_, iron citrate, peptone, and casein hydrolysate (more detail in Table S2). Cultural filtrates were analyzed via reporter assay (more detail in Figure S7), SPE, and HRMS, which revealed that iron availability was the dominant regulatory variable: supplementation with 0.2 mM iron citrate suppressed desferrioxamine E production while activating albomycin δ2 synthesis, whereas iron-limited conditions (only trace iron from media components) triggered sustained desferrioxamine E biosynthesis. This iron-mediated switch reflects a sophisticated metabolic adaptation where the strain dynamically modulates siderophore versus sideromycin production in response to environmental iron levels, prioritizing iron-scavenging desferrioxamine E under iron scarcity and switching to iron-utilizing albomycin δ2 when iron is abundant.

Iron is an essential yet often growth-limiting micronutrient for microorganisms. In bacterial cells, active transport of ferric iron (Fe^3+^) uptake is mediated by siderophores—low-molecular-weight chelators synthesized and secreted by microbes [27]. The specific iron-chelating molecule secreted by the microorganism binds extracellular Fe^3+^, after which the iron–siderophore complex is internalized via membrane transport proteins. Within the cell, a redox reaction reduces iron to the ferrous state, Fe^2+^, while the apo-siderophore is recycled for additional iron scavenging [28,29].

Upon cellular uptake, iron becomes actively incorporated into biochemical pathways, while its excess serves as a regulatory signal through interaction with DmdR proteins (divalent metal-dependent regulators) [30]. These metalloregulatory proteins exhibit high affinity for divalent metal ions, and upon metal binding, undergo conformational changes that enable their function as transcriptional repressors. The Fe^2+^–DmdR complex specifically binds operator sequences of iron-uptake genes (e.g., siderophore biosynthesis clusters), downregulating their expression to maintain intracellular metal homeostasis.

Genomic investigation of the desferrioxamine E and albomycin δ2 BGCs revealed a conserved regulatory element near the promoter region of the desferrioxamine E gene cluster [31,32]. This region contains several palindromic sequences—putative binding sites for the metalloregulatory protein DmdR—located upstream of the *desA* and *desF* genes. This finding led us to hypothesize a regulatory mechanism where iron-bound DmdR may directly repress desferrioxamine biosynthesis by binding to these putative operator sites and indirectly derepress albomycin δ2 production, creating a metabolic switch detectable in the reporter strain *E. coli* JW5503 ΔtolC.

**Figure 5:**
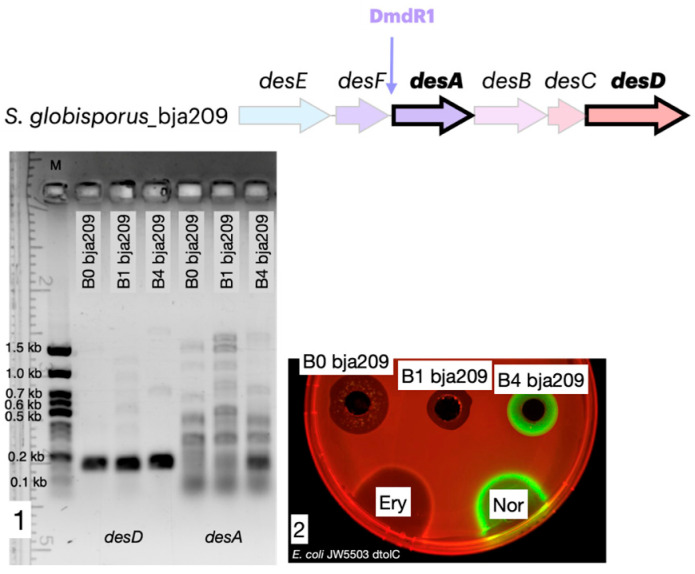
Iron represses *desA* gene expression and desferrioxamine biosynthesis. (**1**) RT-PCR agarose gel analysis demonstrating that *desA* expression (encoding a key biosynthetic enzyme) is repressed in iron-supplemented media (B0, B1) and activated in iron-depleted medium (B4). (**2**) Corresponding antibacterial activity of the culture filtrate against the test strain. Activity is observed only under conditions that induce *desA* expression (B4). The agar plates were spotted with erythromycin, 5 μg (Ery), and norfloxacin, 1 μg (Nor). Katushka2S and TurboRFP signals visualized via ChemiDoc MP with red and green colors, respectively.

To examine the influence of iron ions on gene expression, we performed RT-PCR analysis. Total RNA was isolated and purified from *S. globisporus* bja209 cultures grown under varying iron conditions (media B0, B1, and B4; Table S4), where desferrioxamine E activity was either observed or absent. cDNA was synthesized from the extracted RNA, followed by amplification of target regions within the desferrioxamine biosynthetic gene cluster (Figure 5).

Our transcriptional analysis revealed a distinct differential expression pattern within the desferrioxamine gene cluster under varying iron conditions. While constitutive expression of *desD* (encoding the NRPS-independent siderophore synthetase) was maintained across all culture media, the key biosynthetic gene *desA* (pyridoxal-dependent decarboxylase) exhibited strict iron-dependent regulation. In iron-supplemented media (B0 and B1 containing 0.2 mM iron citrate), complete suppression of *desA* transcription was observed, whereas robust *desA* expression occurred exclusively under iron-limited conditions (B4 medium without iron citrate supplementation). While our data are consistent with a role for a DmdR-like regulator, direct experimental evidence, such as demonstrating protein–DNA binding, is required to unequivocally confirm this mechanism. Notably, identical transcriptional profiles were observed in *S. globisporus* 4-3 (Figure S8), demonstrating conservation of this regulatory paradigm across strains producing structurally distinct desferrioxamine variants.

**Figure 6:**
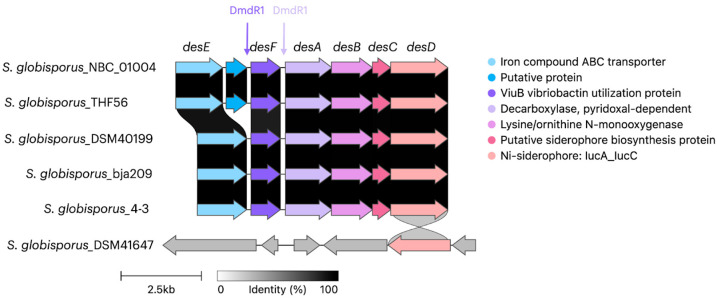
Alignment of desferrioxamine biosynthetic gene clusters (BGCs) across phylogenetically related *S. globisporus* strains. Genes encoding core biosynthetic enzymes (*desA*—decarboxylase; *desB*—monooxygenase; *desC*—acyltransferase; *desD*—synthetase) are highly conserved. Note the presence of an additional ORF of unknown function in some strains. Strains bja209 and 4-3 harbor identical BGCs but produce desferrioxamine E and B, respectively. The desferrioxamine BGC contains sequences encoding a DmdR1 regulator, located upstream of the *desF* and *desA* genes.

As noted previously, the desferrioxamine BGC is highly conserved among streptomycetes and is present in all *S. globisporus* genomes available in GenBank, including both the studied strain bja209 and the previously characterized strain 4-3. While these two strains exhibit 100% homology in their desferrioxamine BGCs, they produce different desferrioxamine variants (E and B, respectively), with only desferrioxamine E demonstrating detectable SOS-inducing and antagonistic activity in our assays. The desferrioxamine BGC is identical across *S. globisporus* bja209, 4-3, and the type strain. In desferrioxamine BGC, only four genes take part in the biosynthesis of target molecules: *desA*—pyridoxal-dependent decarboxylase; *desB*—monooxygenase; *desC*—acetyltransferase; *desD*—siderophore biosynthetic enzyme. The remaining genes are responsible for delivery and reduction of the siderophore molecule: *desE*—lipopolysaccharide receptor ABC transporter; *desF*—ferroxamine-reductase [33] (Figure 6). Notably, the two most phylogenetically proximate strains to bja209 contain an additional ORF of uncharacterized function within this cluster, though its absence in functional producers suggests it plays no essential role in desferrioxamine biosynthesis.

Desferrioxamine E and B are biosynthesized via a common nonribosomal peptide synthetase (NRPS)-independent pathway (NIS) that utilizes identical enzymatic machinery [34]. Both siderophores originate from L-lysine, which undergoes sequential transformations via the action of decarboxylase (DesA) to form cadaverine, followed by hydroxylation catalyzed by monooxygenase (DesB) to yield N-hydroxycadaverine. The key structural divergence arises during the DesC-catalyzed N-acylation reaction, where the N-hydroxycadaverine intermediate undergoes modification by either acetyl-CoA or succinyl-CoA. Subsequent assembly of the desferrioxamine molecules is mediated by the NRPS-independent siderophore synthetase (DesD) [35]. Structural analysis suggests that desferrioxamine E, which induces the SOS response in reporter strains, is composed of N-hydroxy-N-succinylcadaverine residues. In contrast, desferrioxamine B, which shows no antagonistic activity, appears to be a homodimer containing both N-hydroxy-N-succinylcadaverine and N-hydroxy-N-acetylcadaverine units [33].

We hypothesized that the intracellular ratio of succinyl-CoA to acetyl-CoA—regulated by independent metabolic processes—may determine whether desferrioxamine B or E is produced, although this hypothesis could not be experimentally validated within the scope of the current study and will be the subject of future investigations.

### 2.6. Albomycin δ2 BGC and Its Phylogenetic Distribution

The compound albomycin δ2 was first identified in the mid-XX century from *S. griseus* [36]. These compounds were characterized by their broad-spectrum antibacterial activity against both Gram-positive and Gram-negative clinically relevant bacterial pathogens, coupled with their distinctive iron-chelating capabilities. For decades, these dual functional attributes—antimicrobial potency and siderophore activity—served as the primary diagnostic criteria for classifying metabolites produced by diverse *Streptomyces* species as “albomycins.” This phenotypic classification approach led to the widespread but potentially misleading perception that albomycin δ2 production was a common and phylogenetically widespread trait among streptomycetes [37].

The structural characterization of albomycin δ2 was proposed in 1949 from *S. subtropicus* (originally classified as *Actinomyces subtropicus*), and in 2012, the complete BGC was identified in *S. griseus* ATCC 700974 [38,39].

Albomycin δ2 belongs to the group of sideromycins, the structure of which has a particular differentiation into siderophore compounds (three N5-hydroxy-N5-acyl-L-ornithines) that are bound through a serine amino acid residue to the antibacterial part (4′-thioxylofuranosyl pyrimidine) [40]. The sideromycin group exhibits remarkable bacterial cell penetration capability due to its siderophore component, which facilitates ferric iron (Fe^3+^) binding and transport. Initially, the antibacterial effect of albomycin δ2 was attributed to its structural mimicry of respiratory chain intermediates, thereby inhibiting bacterial metabolism [36]; however, studies in 2000 revealed a more sophisticated mechanism of action, demonstrating that albomycin δ2 structurally consists of two distinct moieties: a siderophore portion and an antibiotic component, linked via an amide bond. Upon entry into bacterial cells via the Fhu-regulated iron transport system, albomycin δ2 undergoes enzymatic activation through hydrolysis of its amide bond by the intracellular peptidase PepN. This cleavage releases the active antibiotic moiety SB-217452 (4′-thioxylofuranosyl pyrimidine), which specifically inhibits seryl-tRNA synthetase [41]. This sophisticated mode of action exemplifies the “Trojan horse” effect in antimicrobial agents.

**Figure 7:**
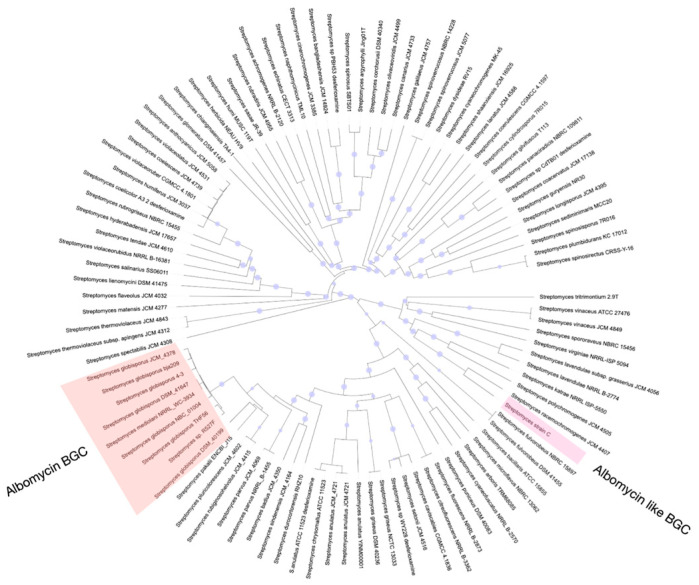
Phylogenetic tree of neighborhood strains demonstrating distribution of albomycin BGCs within the *S*. *globisporus* clade. The maximum-likelihood phylogenetic tree, based on whole-genome sequences, shows the evolutionary relationships between various *Streptomyces* type strains. The presence (filled circles) or absence (empty circles) of a complete albomycin BGC is indicated at the tips of the branches. The cluster is exclusively found in a monophyletic group of *S. globisporus* strains (highlighted in red), contradicting the previous assumption of its broad distribution across the genus.

During our screening of antibacterial compounds, we reisolated and identified an albomycin-producing strain belonging to *S. globisporus*. Notably, none of the several thousand other antibiotic-producing strains we tested demonstrated albomycin δ2 production capability, contradicting the previous assumption that this molecule is widely synthesized among streptomycetes. To investigate this discrepancy, we analyzed publicly available whole-genome sequences of *Streptomyces* species in GenBank for the presence of BGCs associated with albomycin δ2 production. Our genomic analysis revealed that BGCs containing genes essential for albomycin δ2 biosynthesis are present only in certain strains of *S. globisporus* (Figure 7), demonstrating a much more restricted phylogenetic distribution than previously believed.

As described above, the reference albomycin δ2 BGC was researched and studied in more detail in the *Streptomyces* sp. strain ATCC700974. Information about the whole genome was not published in GenBank, which restricts us from conducting phylogenetic analysis and identifying the species affiliation of the strain. The strain ATCC700974 was originally isolated and deposited in the international culture collection by S. Waksman and colleagues, who classified it phenotypically as *S. griseus* in their publications. However, according to modern taxonomic criteria, this strain most likely belongs to *S. globisporus*.

**Figure 8:**
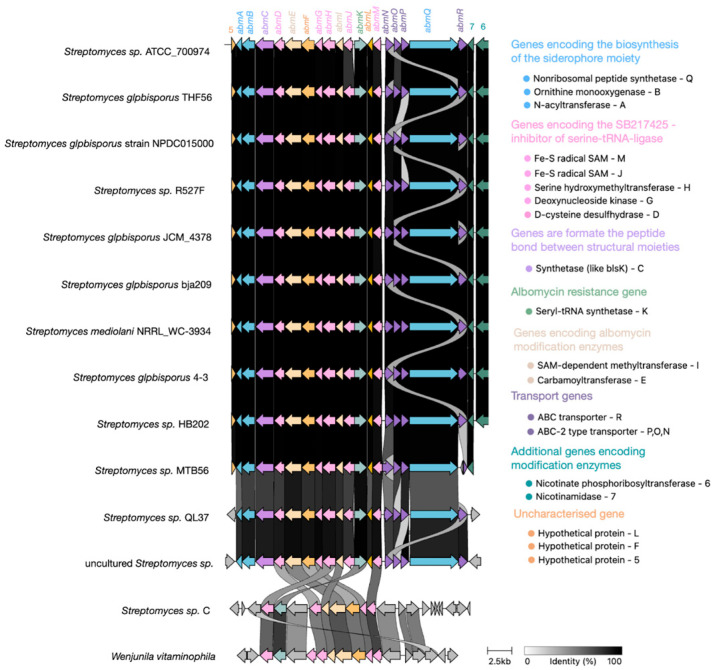
Evolution of the albomycin δ2 BGC within the *S. globisporus* clade. A comparative alignment map of the albomycin BGCs across related *S. globisporus* strains reveals high conservation of genes encoding enzymes responsible for synthesizing the core antibiotic structure (highlighted in blue and pink). In contrast, genes encoding transporters (highlighted in purple), resistance genes (highlighted in green), and additional biosynthetic genes (ivory, turquoise, and light purple) exhibit significant variability and duplication events, suggesting possible adaptation to different ecological niches and mechanisms of self-resistance.

Alignment of all available albomycin δ2 BGCs (Figure 8) revealed high sequence homology among core biosynthetic genes, with minor variations observed primarily in transporter genes, likely reflecting adaptive diversification. Notably, the genes *albR* and *albN*, as well as *albP* and *albO*, appear to have evolved through gene duplication events from common ancestral sequences followed by functional divergence, resulting in structurally distinct but functionally related gene pairs.

**Figure 9:**
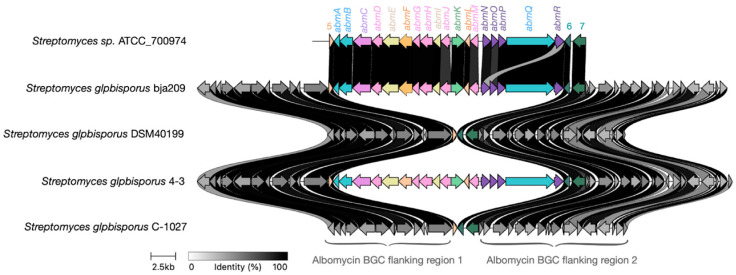
Genomic context comparison supports horizontal acquisition of the albomycin BGC. Alignment of the chromosomal region containing the albomycin biosynthetic gene cluster (BGC) in producer strains versus the homologous empty site in non-producer strains of *S. globisporus*. The high conservation of syntenic flanking genes (gray arrows) surrounding either the complete albomycin BGC (colored arrows) or an empty insertion site provides that the cluster was acquired through a single horizontal gene transfer (HGT) event, rather than through gradual, in situ evolution within the species.

Notably, Figure 9 reveals partially degraded, non-functional albomycin δ2 BGCs in phylogenetically confirmed *S. globisporus* strains MTB56 and QL37. Furthermore, GenBank contains *S. globisporus* genomes that completely lack the albomycin δ2 BGC while retaining clear homologous sequences flanking the cluster’s insertion site in producing strains. These observations suggest the albomycin δ2 cluster likely originated in an ancestral precursor and was subsequently acquired by an *S. globisporus* strain through horizontal gene transfer, followed by minor mutations primarily affecting transporter genes while preserving the core biosynthetic architecture (Figure 9).

The genomic evidence—specifically, the presence of empty, conserved attachment sites in non-producers—strongly suggests a single HGT event followed by differential preservation. We hypothesize that the cluster is retained in specific strains because it confers a significant ecological advantage, likely in competitive microniches where inhibition of bacterial competitors via albomycin production is crucial for survival. In other strains, the energetic cost of maintaining the large BGC may have led to its loss through genetic drift, especially if these strains occupy niches where the antibiotic provides no selective benefit.

As previously described, salmycins and ferrimycins represent additional members of the sideromycin class [42,43]. These molecules contain structural moieties that, upon enzymatic hydrolysis, release inhibitors targeting seryl-tRNA synthetase, though their chemical architectures differ substantially from albomycin δ2. Notably, their biosynthetic gene clusters remain uncharacterized in the published literature. The sideromycin family further includes microcin C, which inhibits aspartyl-tRNA synthetase, and agrocin 84 targets leucyl-tRNA synthetase [44].

Self-resistance to sideromycins is mediated by efflux transporter genes located within their BGC. The albomycin δ2 BGC exhibits a unique feature—it contains an additional gene of seryl-tRNA synthetase (SerRS) resistant to SB-217452. Phylogenetic analysis revealed this cluster-associated SerRS was evolutionarily distant from the housekeeping SerRS found in streptomycetes, with NCBI BLAST (version 2.17.0) indicating its origin likely traces to rare actinomycete genera. Notably, the active site motifs of housekeeping SerRS in *S. globisporus* strains (both albomycin δ2 producers and non-producers) show no adaptive mutations, suggesting the resistant paralog is essential for self-protection.

The original researchers who characterized the albomycin δ2 BGC proposed that such duplicate SerRS genes could serve as markers for discovering novel tRNA synthetase inhibitors. They identified a GenBank-deposited strain (Figure 8) phylogenetically distant from *S. globisporus* that harbors a SerRS homolog resembling the albomycin-resistant variant and partial albomycin-like biosynthetic genes (though lacking key components). While this suggests potential production of an uncharacterized tRNA synthetase inhibitor, experimental validation remains lacking. The cluster may represent an ancestral albomycin-like insertion event, analogous to what occurred in *S. globisporus*. Our genomic surveys identified similar duplicate SerRS genes in some *S. anulatus* strains (a *S. globisporus* relative), though without accompanying albomycin δ2 biosynthetic genes. This implies these clusters may produce distinct, yet-to-be-identified tRNA synthetase inhibitors.

## 3. Discussion

The *S. globisporus* bja209 strain was identified in a screen of natural actinomycete isolates against the *E. coli* JW5503 ΔtolC DualRep2(c) strain and exhibits distinct antibacterial activity on different growth media (ISP3/ISP6). HPLC-MS analysis coupled with genomic context examination identified three active metabolites: albomycin δ2, desferrioxamine E, and C-1027.

Although antagonistic activity of desferrioxamine E was previously reported, our reporter system revealed its specific mechanism of action via disruption of DNA synthesis. Notably, the structurally similar desferrioxamine B (produced by another strain we studied) lacked this activity. Desferrioxamine E demonstrated growth inhibition against carbapenem-resistant *A. baumannii* blaNDM and the phytopathogen *C. michiganensis*. Its production was suppressed in media containing 0.2 mM Fe^3+^; however, the exact cellular target responsible for SOS response induction requires further investigation. Bioinformatic analysis showed widespread distribution of desferrioxamine BGCs among *S. globisporus* and related species, and they are present in all representatives of this taxon. In contrast, the albomycin δ2 BGC appears to be restricted to *S. globisporus* and is found in only 80% of its genomes. The albomycin δ2 cluster likely originated outside this taxon, as published genomes show no evidence of gradual cluster evolution but rather clear homologous flanking regions where the albomycin δ2 cluster is either present or absent.

This study has elucidated patterns of secondary metabolite cluster distribution within the *S. globisporus* taxon and their regulation by environmental iron concentrations.

## 4. Materials and Methods

The strain bja209 was isolated from the A1 humus horizon of a chernozem soil (the pH was 6.0, the sample was carbonate-free, and it contained 3% organic matter). No effervescence was observed upon addition of hydrochloric acid collected in the Republic of Khakassia (51°42′23.8′′ N 88°20′10.1′′ E). Sample collection was performed according to a previously described methodology [45]: a specimen from the A1 humus layer (10–15 cm depth) was collected using a sterile spatula and placed in a sterile sample container [46]. Actinobacteria were isolated by surface-plating ten-fold serial dilutions of soil suspensions onto oatmeal agar (ISP3) [46,47] supplemented with nystatin (250 μg/mL) and nalidixic acid (10 μg/mL) to suppress fungal and Gram-negative bacterial growth, respectively. Additionally, rubomycin (5 μg/mL) was employed as a selective marker to isolate rare members of the microbial community [48,49]. The plates were incubated for 14 days at 28 °C.

The strain bja209 was isolated into pure culture from the primary plating along with other mycelial actinobacteria based on morphological characteristics. The strain was maintained on ISP3 and ISP6 agar media [50].

Cultural characteristics of the strain bja209 (including aerial mycelia coloration and soluble pigment excretion) were observed on solid media recommended by the International Streptomyces Project after cultivation for up to 14 days at 28 °C [51].

Morphological characteristics of the spore’s chains and the spore surface of strain bja209 were detected using a JSM-6380LA scanning electron microscope (JEOL Ltd., Akishima, Tokyo, Japan) following 4 days of growth at 28 °C on organic agar 79 medium.

Carbon source utilization (mono- and oligosaccharides, alcohols) was assessed using the disc-diffusion method (HiMedia Laboratories, Maharashtra, India) on basal medium ISP9 mineral with 0.04% bromocresol purple, as a pH indicator, at 28 °C for 14 days. Enzyme activities (starch, cellulose, casein) were detected according to protocols by measuring hydrolysis zone diameters [44].

Antibiotic susceptibility was determined by the disk-diffusion method. A spore suspension of the strain bja209 (5 McFarland standard) was spread on ISP3 solid media. After drying, antibiotic-impregnated discs (HiMedia Laboratories Pvt. Ltd., Maharashtra, India) were applied, and plates were incubated for 7 days at 28 °C, followed by measurement of inhibition zones.

Genomic DNA of the strain bja209 was isolated using a previously described methodology [50]. The genome of the strain bja209 was sequenced de novo using the SURFSeq 5000 platform (GeneMind, Shenzhen, China). Genome assembly was performed using Shovill [52] (Version 1.1.0) with the SPAdes assembler option. The assembly quality and completeness, assessed by BUSCO v5.8.0 with the streptomycetales_odb10 dataset, was C:99.6% [S:99.4%, D:0.1%], F:0.4%, M:0.1% (n:1579). The total length of the assembly was 8,031,129 bp [53]. Genome annotation was performed using PROKKA version 1.14.6 [54]. Analysis of biosynthetic gene clusters (BGCs) for antibiotics was conducted using [20].

Phylogenetic affiliation was determined using whole-genome sequencing data using the Type (Strain) Genome Server (TYGS) [55]. The strain genome was automatically compared against all type strain genomes available in the TYGS database using the MASH algorithm [56].

Phylogenetic reconstruction was performed using FastME 2.1.6.1 based on Genome BLAST (version 2.17.0) Distance Phylogeny (GBDP) distances calculated from whole-genome nucleotide sequences. Branch lengths were scaled according to the GBDP d5 distance formula [57].

The antibacterial activity of the bioactive compounds was tested against the bacterial strain *E. coli* JW5503 ΔtolC, characterized by Δ*tolC* gene deletion that disturbs the efflux pump, and the strain *E. coli* BW25113 lptD, featuring deletion in the *lptd* gene (amino acid residues 330–352) leading to impaired lipopolysaccharide layer synthesis and consequently increased permeability to low-molecular-weight compounds [4]. These strains harbor the plasmid vector pDualrep2, which encodes the fluorescent proteins TurboRFP and Katushka2S. Expression of these reporter proteins is induced by sublethal concentrations of compounds that trigger the SOS response or inhibit translation, respectively. Fluorescence signals were quantified 24 h post-treatment using a ChemiDoc MP imaging system (Bio-Rad, Hercules, CA, USA) with predefined optical channels (Cy3 and Cy5 filters), enabling selective detection of TurboRFP and Katushka2S emission spectra.

Screening on the reporter strain was performed using an agar diffusion assay as previously described [45]. The strain bja209 was cultured on ISP3 and ISP6 media, with testing conducted on days 3, 6, 9, and 12 of growth. Agar blocks (5 mm diameter) were aseptically excised from sporulated colonial lawns and placed onto LB agar plates pre-inoculated with the appropriate test organism. Crude extracts from bja209 were evaluated against opportunistic pathogens, multidrug-resistant (MDR) ESKAPE group isolates, and clinical isolates. Test bacterial lawns were maintained on Mueller–Hinton (MH) agar (HiMedia Laboratories, Maharashtra, India), while yeast strains were maintained on glucose–peptone–yeast (GPY) agar (HiMedia Laboratories, Maharashtra, India) [58]. All plates were incubated at 37 °C for 24 h, after which inhibition zone diameters were measured.

For detailed bioactivity studies, the strain bja209 was cultured in liquid ISP3 and ISP6 at 28 °C for 5–14 days under continuous agitation (200 rpm) using a New Brunswick Innova44 orbital shaker (Eppendorf, Framingham, MA, USA). Cells were removed from the culture broth by centrifugation at 4000× *g*. The resulting supernatant was concentrated and purified via solid-phase extraction (SPE) using Poly-Prep Econo-Pac chromatography columns (Bio-Rad, Hercules, CA, USA) packed with 1 mL of LPS-500H sorbent (Technosorbent, Moscow, Russia). Elution was performed with a step gradient of water–acetonitrile, followed by fraction collection. All fractions were subsequently screened for antagonistic activity using the agar diffusion method described above.

Subsequently, active fractions were purified using an Agilent 1260 series HPLC system (Hewlett-Packard-Strasse 8, 76337 Waldbronn, Germany) equipped with a diode array detector and automated fraction collector. Separation was achieved on a Luna 5 μm C18(2) 100 Å column (250 × 4.6 mm; Phenomenex, Torrance, CA, USA) with a water–acetonitrile gradient. Detailed chromatographic parameters for each compound are provided in the Supplementary Materials.

Active fractions were analyzed using a chromatographic–mass spectrometric system consisting of an UltiMate 3000 UHPLC system (Thermo Fisher Scientific, Waltham, MA, USA), an Acclaim RSLC 120 C18 column (2.2 μm, 2.1 × 100 mm; Thermo Fisher Scientific, USA), and a maXis II 4G ETD qTOF mass spectrometer (Bruker Daltonics, Bremen, Germany). The mass spectrometer operated in electrospray ionization (ESI) mode with the following parameters: full scan range of 100–1500 m/z, MS/MS mode (top 3 most intense ions selected for fragmentation), collision-induced dissociation (CID) energy (10–40 eV). Mass spectral data were processed using OpenChrom Lablicate Edition (v1.4.0.202201211106) and TOPPView (v3.3.0) [59]. Chemical structures were identified using the GNPS (Global Natural Products Social Molecular Networking) database [60], NPAtlas [61,62], and Dictionary of Natural Products 31.1.

## Figures and Tables

**Table 1:** Bja209 antibiotic resistance profile using standardized disc-diffusion assays.

Type of Antibiotic	Bja209 Strain (Size of Lysis Zone, mm)	Interpretation (CLSI) [25]
Aminoglycosides		
Amikacin, 30 μg (АК 30)	33	Susceptible
Gentamicin, 10 μg (CN 10)	20	N/A
Kanamycin, 50 μg (KM 50)	27	N/A
Streptomycin, 2000 μg (S 2000)	–	Resistant
Steptothricin, 72 μg (ST 72)	–	Resistant
Tobramycin, 10 μg (TOB 10)	22	Susceptible
Beta-lactams/Beta-lactams + Beta-lactamase Inhibitor		
Aztreonam, 30 μg (ATM 30)	–	Resistant
Amoxicillin/clavulanic acid, 30 μg (AMC 30)	10	Intermediate
Ampicillin, 100 μg (AMP 100)	–	Resistant
Meropenem, 15 μg (MEM 15)	–	Resistant
Oxacillin, 5 μg (Ox 5)	–	Resistant
Penicillin, 100 μg (PEN 100)	–	Resistant
Tazobactam, 110 μg (TPZ 110)	–	Resistant
Cephalosporins		
Cefoxitin, 30 μg (FOX 30)	–	Resistant
Ceftazidime, 30 μg (CAZ 30)	–	Resistant
Sulfonamides		
Trimethoprim, 25 μg (TMP 25)	–	Resistant
Trimethoprim-Sulfamethoxazole, 25 μg (SXT 25)	–	Resistant
Fluoroquinolones		
Levofloxacin, 5 μg (Le5)	20	Susceptible
Norfloxacin, 10 μg (NOR 10)	–	Resistant
Ciprofloxacin, 5 μg (CIP 5)	13	Susceptible
Other		
Vancomycin, 30 μg (Va 30)	15	N/A
Clindamycin, 2 μg (DA 2)	27	Susceptible
Erythromycin, 15 μg (Е15)	16	Susceptible
Rubomycin, 5 μg (RB 5)	–	Resistant

## Data Availability

The original contributions presented in this study are included in the article. Further inquiries can be directed to the corresponding author.

## Supplementary Materials

The following supporting information can be downloaded at https://www.mdpi.com/article/10.3390/molecules30193871/s1: Table S1: Comparative genomic analysis of *Streptomyces globisporus* bja209 and related type strains; Table S2: Cultural characteristics of *Streptomyces globisporus* strains bja209 (1), *S. globisporus* DSM 40199 (2), and *S. globisporus* subsp. *globisporus* 4-3 (3) on ISP media; Table S3: Carbohydrate utilization profiles of *Streptomyces globisporus* strains bja209 (1), *S. globisporus* subsp. *globisporus* DSM 40199 (2), and *S. globisporus* subsp. *globisporus* 4-3 (3) on ISP medium 9; Figure S1: The multiple sequence alignment of β-galactosidase enzymes from closely related *S. globisporus* strains; Figure S2: The alignment of nucleotide sequences from the araR repressor regulatory region reveals critical features governing arabinose metabolic regulation; Table S4: Antibacterial activity spectrum of *S. globisporus* bja209 against clinically relevant strains. Inhibition zone diameters (mm) are shown for albomycin and desferrioxamine E; Figure S3: HPLC chromatogram showing the purification of albomycin δ2; Figure S4: HPLC analysis of the deferoxamine E-containing fraction; Figure S5: Detection of iron-chelating activity of deferoxamine E using the ChromeAzurol S (CAS) assay; Figure S6: Antibacterial activity of desferrioxamine E against antibiotic-resistant *E. coli* strains; Table S5: Composition of modified media used to identify nutritional inducers of deferoxamine E and albomycin production in *S. globisporus* bja209; Figure S7: Activity of crude broth of bja209 against *E. coli* JW5503 ΔtolC and *E. coli* BW25113 lptD; Figure S8: Analysis of gene expression in *S. globisporus* strains bja209 and 4-3 under iron-dependent conditions by RT-PCR. References [63,64,65,66] are cited in the Supplementary Materials.

## Abbreviations

The following abbreviations are used in this manuscript:

BGC	Biosynthetic Gene Cluster
ISP	International Streptomyces Project
