# Supplementary material for: The Regulation of the Albomycin and Desferrioxamine E Biosynthesis in Streptomyces globisporus bja209
Source: Molecules. 2025 Sep 24;30(19):3871. doi: 10.3390/molecules30193871 (PMC12525621; doi:10.3390/molecules30193871)
Supplement: Supplementary file 1 [file molecules-30-03871-s001.zip › molecules-3845046-supplementary.pdf]

Supplementary materials

Table S1. Comparative genomic analysis of *Streptomyces globisporus* bja209 and related type strains. Values include genome size, GC content, number of genes/contigs/rRNA/tRNA, Average Nucleotide Identity (ANI), digital DNA–DNA hybridization (dDDH), and GC content difference.

Strains: (1) bja209; (2) *S. globisporus* NBC_01004 (GCF_045162705.1); (3) *S. globisporus* THF56 (CP029361.1); (4) *S. globisporus* DSM 40199 (GCA_014649555.1); (5) *S. globisporus* C-1027 (GCF_000261345.2); (6) *S. globisporus* 4-3 (GCA_935184445.1).

Genome features	1	2	3	4	5	6
Genome size, mbp	8,074,519	7,957,062	7,666,521	7,783,599	7,783,599	7,914,357
G+C content, %	71.42	71.43	71.54	71.54	71.54	71.54
Amount of genes	7186	7074	6837	6921	6921	7040
Amount of contigs	2	2	3	3	3	62
Amount of rRNA	18	18	18	18	18	3
Amount of tRNA	83	84	84	79	79	83
ANI with bja209, %	--	99.49	99.48	96.01	96.01	99.40
dDDH, %	--	96.00	95.90	82.00	82.00	92.50
G+C content difference, %	–	0.02	0.09	0.10	0.10	0.13

Table S2. Cultural characteristics of *Streptomyces globisporus* strains bja209 (1), *S. globisporus* DSM 40199 (2), and *S. globisporus* 4-3 (3) on ISP media.

Observations include growth intensity, aerial and substrate mycelium color, and soluble pigment production after 14 days of incubation at 28°C. Abbreviations: ISP, International *Streptomyces* Project media; +, growth; –, no growth.

	1	2	3
ISP2			
Growth	+	+	+
Aerial spore-mass color	white	ocher	oyster white
Substrate mycelial color	white	ocher	beige
Soluble pigment	none	none	none
ISP3			
Growth	+	+	+
Aerial spore-mass color	sand olive	beige	oyster white
Substrate mycelial color	oyster-beige	oyster beige	brown beige
Soluble pigment	none	non	sand yellow
ISP4			
Growth	+	+	+
Aerial spore-mass color	oyster-beige	light grey	white
Substrate mycelial color	beige	green-brown	colorless
Soluble pigment	none	none	none
ISP5			
Growth	+	+	+
Aerial spore-mass color	oyster-beige	colorless	light olive
Substrate mycelial color	sand	oyster-beige	sand yellow

Soluble pigment	none	none	sand yellow
ISP6			
Growth	+	+	+
Aerial spore-mass color	white	colorless	white
Substrate mycelial color	beige	oyster-beige	beige
Soluble pigment	none	none	none
ISP7			
Growth	+	+	+
Aerial spore-mass color	oyster-beige	colorless	oyster-beige
Substrate mycelial color	beige	sand yellow	yellow-red
Soluble pigment	none	none	none

Table S3. Carbohydrate utilization profiles of *Streptomyces globisporus* strains bja209 (1), *S. globisporus* DSM 40199 (2), and *S. globisporus* 4-3 (3) on ISP medium 9. Carbon source utilization was assessed using the disc-diffusion method on basal mineral medium supplemented with bromocresol purple (0.04%) as a pH indicator. Symbols: +, utilization (acid production); –, no utilization. Abbreviations: ISP, International *Streptomyces* Project.

Carbone source	1	2	3
D-glucose (De)	+	+	+
Fructose (Fc)	+	+	+
Mannose (Mo)	+	no data	+
Xylose (Xy)	+	no data	+
Cellobiose (Ce)	+	+	no data
Trehalose (Te)	+	no data	no data
Rhamnose (Rh)	+	+	+
Lactose (La)	+	no data	–
Maltose (Ma)	+	no data	+
Mannitol (Mn)	+	+	+
Arabinose (Ar)	–	+	+
Galactose (Ga)	–	no data	+
Inositol (Is)	–	no data	–
Sucrose (Su)—invertase	–	no data	–
Inulin (In)—inulinases	–	no data	–
Melibiose (Mb)—alpha-galactosidase	–	no data	–
Sorbitol (Sb)	–	no data	–
Salicin (Sa)	–	no data	no data
Adonitol (Ad)	–	no data	–
Dulcitol (Du)	–	no data	–
Raffinose (Rf)	–	–	–

Figure S1. The multiple sequence alignment of β-galactosidase enzymes from closely related *S. globisporus* strains confirms the conservation of the catalytic site. The alignment demonstrates that key catalytic residues Glu160 and Glu358 (highlighted in red) are identical across all strains, indicating essential roles in the enzyme's function that have been preserved through evolution.

```
LacZ3_bja209      MPLTHLDLAGHPRPQLVREADWHDLSGPWQFAFDDADLGRSERCMDPSVTAPYTDVVTVPYPPESKASGIADSGHHPVLW
LacZ3_4-3         --------------------------------------------------------------------------------
LacZ3_DSM40199    MPLTHLDLAGHPRPQLVREADWHDLSGPWQFAFDDADLGRSERWMDPSVLAPYTDVVTVPYPPESKASGIADSGHHPVLW
LacZ3_41647       MPLTHLDLAGHPRPQLVREADWHDLSGPWQFAFDDADLGRSERWMDPSVTAPYTDVVTVPYPPESKASGIADSGHHPVLW
LazZ3_NCB01004    MPLTHLDLAGHPRPQLVREADWHDLSGPWQFAFDDADLGRSERWMDPSVLAPYTDVVTVPYPPESKASGIADSGHHPVLW
LacZ3_THF56       --------------------------------------------------------------------------------
                                                                                                  80
LacZ3_bja209      YRRTLALSAPADGHRLLLHFGAVDYRAEVWLDGRLVGRHEGGHTGSSCDLTDAVRHGAEQVLVVRAEDQPLDAAQPRGKQ
LacZ3_4-3         --------------------------------------------------------------------------------
LacZ3_DSM40199    YRRTLALSAPADGHRLLLHFGAVDYRAEVWLDGRLVGRHEGGHTGFSCDLTDAVRHGAEQVLVVRAEDQPLDAAQPRGKQ
LacZ3_41647       YRRTLALSAPADGHRLLLHFGAVDYRAEVWLDGRLVGRHEGGHTGFSCDLTDAVRHGAEQVLVVRAEDQPLDAAQPRGKQ
LazZ3_NCB01004    YRRTLALSAPADGHRLLLHFGAVDYRAEVWLDGRLVGRHEGGHTGFSCDLTDAVRHGAEQVLVVRAEDQPLDAAQPRGKQ
LacZ3_THF56       --------------------------------------------------------------------------------
                                                                                                 160
LacZ3_bja209      DWRERPHVIWYHRTSGIWQPVWLEEVPDQHLTTLHWTPDVAHARVRCALRLNRWPRRTLRTDVRPVLGDRVLAEQRVLAE
LacZ3_4-3         --------------------------------------------------------------------------------
LacZ3_DSM40199    DWRERPHVIWYHRTSGIWQPVWLEEVPDQHLTTLHWTPDVAHARVRCALRLNRWPRRTLRTDVRPVLGDRVLAEQRVLAE
LacZ3_41647       DWRERPHVIWYHRTSGIWQPVWLEEVPDQHLTTLHWTPDVAHARVRCALRLNRWPRRTLRTDVRLVLGDRVLAEQRVLAE
LazZ3_NCB01004    DWRERPHVIWYHRTSGIWQPVWLEEVPDQHLTTLHWTPDVAHARVRCALRLNRWPRRTLRTDVRLVLGDRVLAEQRVLAE
LacZ3_THF56       --------------------------------------------------------------------------------
                                                                                                 240
LacZ3_bja209      DQETVFDIAVPALRHAQDLDALLWSPESPRLVDATVVITDAEDGHEIDRVTSYLGLRDIGWEDGGFQLNHKPCFLRLALQ
LacZ3_4-3         --------------------------------------------------------------------------MRLALQ
LacZ3_DSM40199    DQETVFDIAVPALRHAQDLDALLWSPESPRLVDATVVITDAEDGHEIDRVTSYLGLRDIGWEDGGFQLNHKPCFLRLALQ
LacZ3_41647       DQETVFDIAVPALRHAQDLDALLWSPESPRLVDATVVITDAEDGHEIDRVTSYLGLRDIGWEDGGFQLNHKPCFLRLALQ
LazZ3_NCB01004    DQETVFDIAVPALRHAQDLDALLWSPESPRLVDATVVITDAEDGHEIDRVTSYLGLRDIGWEDGGFQLNHKPCFLRLALQ
LacZ3_THF56       --------------------------------------------------------------------------MRLALQ
                                                                                                 320
LacZ3_bja209      QGYWPDSHLAASADELRRDAELAKEAGLNGLRVHQKLEDPRFLYWADRLGLMLWAEMPSAFAFGTVTMERVVREWTEAVS
LacZ3_4-3         QGYWPDSHLAASADELRRDAELAKEAGLNGLRVHQKLEDPRFLYWADRLGLMLWAEMPSAFAFGTVTMERVVREWTEAVS
LacZ3_DSM40199    QGYWPDSHLAASADELRRDAELAKEAGLNGLRVHQKLEDPRFLYWADRLGLMLWAEMPSAFAFGTVTMERVVREWTEAVS
LacZ3_41647       QGYWPDSHLAASADELRRDAELAKEAGLNGLRVHQKLEDPRFLYWADRLGLMLWAEMPSAFAFGTVTMERVVREWTEAVS
LazZ3_NCB01004    QGYWPDSHLAASADELRRDAELAKEAGLNGLRVHQKLEDPRFLYWADRLGLMLWAEMPSAFAFGTVTMERVVREWTEAVS
LacZ3_THF56       QGYWPDSHLAASADELRRDAELAKEAGLNGLRVHQKLEDPRFLYWADRLGLMLWAEMPSAFAFGTVTMERVVREWTEAVS
                                                                                                 400
LacZ3_bja209      RDLSHPSIVAWVPVNESWSTPNPALVPQQRHFIDSLYHLTKALDPSRPAVSNDGWEISAADIWGVHDYTQQASVLRERYG
LacZ3_4-3         RDLSHPSIVAWVPVNESWSTPNPALVPQQRHFIDSLYHLTKALDPSRPAVSNDGWEISAADIWGVHDYTQQASVLRERYG
LacZ3_DSM40199    RDLSHPSIVAWVPVNESWSTPNPALVPQQRHFIDSLYHLTKALDPSRPAVSNDGWEISAADIWGVHDYTQQASVLRERYG
LacZ3_41647       RDLSHPSIVAWVPVNESWSTPNPALVPQQRHFIDSLYHLTKALDPSRPAVSNDGWEISAADIWGVHDYTQQASVLRERYG
LazZ3_NCB01004    RDLSHPSIVAWVPVNESWSTPNPALVPQQRHFIDSLYHLTKALDPSRPAVSNDGWEISAADIWGVHDYTQQASVLRERYG
LacZ3_THF56       RDLSHPSIVAWVPVNESWSTPNPALVPQQRHFIDSLYHLTKALDPSRPAVSNDGWEISAADIWGVHDYTQQASVLRERYG
                                                                                                 480
LacZ3_bja209      HASLRQGEFTDPWPGAKRLLLPGVLHQGQPVVLSEFGGTTFEPAEGEAWFGYDTVVTREEYAERLSSLVEAAVDSGLAGF
LacZ3_4-3         HASLRQGEFTDPWPGAKRLLLPGVLHQGQPVVLSEFGGTTFEPAEGEAWFGYDTVVTREEYAERLSSLVEAAVDSGLAGF
LacZ3_DSM40199    HASLRQGEFTDPWPGAKRLLLPGVLHQGQPVVLSEFGGTTFEPAEGEAWFGYDTVVTREEYAERLSSLVEAAVDSGLAGF
LacZ3_41647       HASLRQGEFTDPWPGAKRLLLPGVLHQGQPVVLSEFGGTTFEPAEGEAWFGYDTVVTREEYAERLSSLVEAAVDSGLAGF
LazZ3_NCB01004    HASLRQGEFTDPWPGAKRLLLPGVLHQGQPVVLSEFGGTTFEPAEGEAWFGYDTVVTREEYAERLSSLVEAAVDSGLAGF
LacZ3_THF56       HASLRQGEFTDPWPGAKRLLLPGVLHQGQPVVLSEFGGTTFEPPEGEAWFGYDTVVTREEYAERLSSLVEAAVDSGLAGF
                                                                                                 560
LacZ3_bja209      CFTQFTDTEQETNGLFTADRTPKLPPAELRAILTRPRRG
LacZ3_4-3         CFTQFTDTEQETNGLFTADRTPKLPPAELRAILTRPRRG
LacZ3_DSM40199    CFTQFTDTEQETNGLFTADRTPKLPPAELRAILTRPRRG
LacZ3_41647       CFTQFTDTEQETNGLFTADRTPKLPPAELRAILTRPRRG
LazZ3_NCB01004    CFTQFTDTEQETNGLFTADRTPKLPPAELRAILTRPRRG
LacZ3_THF56       CFTQFTDTEQETNGLFTADRTPKLPPAELRAILTRPRRG
                                                        599
```

Figure S2. Key regulatory features in the araR repressor promoter region revealed by nucleotide sequence alignment. The alignment identifies conserved operator sites (underlined) for AraR repressor binding and essential promoter elements (-10/-35, bold). Single-nucleotide polymorphisms (blue) are located within these functional regions, suggesting potential impact on arabinose metabolism regulation [1].

```
Bja209_p_araR3    GCCGGT----GAGATTGCAGCAATCAATCTCTTGCGTCAACCACTTGGCGGTGCTTTTGTAACCCCAGCGTCAAGAAATG
4-3_p_araR3       CTTCGAGGTCCGGGACGCCGCTCCGGATTTCCTTCATCGCATTCTCCCACCCAATTCATTCTCTTAAAAGGAATGAATTT
DSM40199_p_araR3  CTTCGAGGTCCGGGACGCCGCTCCGGATTTCCTTCATCGCATTCTCCCACCCAATTCATTCCCTTGAAAGGAATGAATTT
                                                     1        -10                    -35

Bja209_p_araR3    CGGAAATTCATTCCTTTTAAGGGAATGAATTGGGTGGGAGAA--TGCGATGAAGGAAATCCGGAGCGGCGTCCCGGACCT
4-3_p_araR3       CCGCATTTCTTGACGCTGG-GGTTACAAA-AGCACCGCCAAGTGGTTGACGCAAGAGATTGATTGCTGCAATCTC----A
DSM40199_p_araR3  CCGCATTTCTTGACGCTGG-GGTTACAAA-AGCACCGCCAAGTGGTTGACGCAAGAGATTGATTGCTGCAATCTC----A

Bja209_p_araR3    CGAAG
4-3_p_araR3       CCGGC
DSM40199_p_araR3  CCGGC
```

Table S4. Antibacterial activity spectrum of *S. globisporus* bja209 against clinically relevant strains.

Inhibition zone diameters (mm) are shown for albomycin and desferrioxamine E. Test strains include multidrug-resistant clinical isolates and reference strains. Abbreviations: MDR, multidrug-resistant; MRSA, methicillin-resistant *Staphylococcus aureus*; ESKAPE pathogens (*Enterococcus faecium*, *Staphylococcus aureus*, *Klebsiella pneumoniae*, *Acinetobacter baumannii*, *Pseudomonas aeruginosa*, *Enterobacter spp.*).

Type of strain		
	Albomycin δ2	Desferrioxamine E
K. pneumoniae blaVIM-1	15	–
K. pneumoniae BlaOXA-48	11	–
K. pneumoniae blaKPC-3	16	–
K. pneumoniae blaOXA-244	18	–
K. pneumoniae blaNDM-1+blaOXA-48	18	–
K. pneumoniae blaNDM-1	15	–
K. pneumoniae blaNDM-5	11	–
K. pneumoniae ATCC 700603	12	–
E.coli mcr-1.1	18	–
E.coli blaVIM-1	18	–
E.coli ATCC 35218	18	–
E.coli ATCC 25922	11	–
Acinetobacter baumannii blaOXA-23+ blaOXA-72	–	–
A. baumannii blaOXA-23	–	–
A. baumannii blaOXA-72	–	–
A. baumannii blaNDM	–	12
Enterococcus faecium	–	–
S. aureus ORGID 90449	10	–
S. aureus MRSA	11	–
S. aureus ATCC 29213	11	–
S. aureus INA 00761	21	–
Pseudomonas aeruginosa blaGES-5	–	–
P. aeruginosa blaVIM	–	–
P. aeruginosa blaVIP+blaIMP	–	

P. aeruginosa ATCC 27853	–	–
M. smegmatis	–	17
C. albicans	–	–
Erwinia amylavora	15	20
Clavibacter michiganensis	15	20
K. pneumoniae 107210323	19	–
K. pneumoniae 53130623	20	–
K. pneumoniae 77270223	21	–
K. pneumoniae 60130323	22	–
K. pneumoniae 152310323	23	–
K. pneumoniae 12-13-14040423	24	–
K. pneumoniae 83180423	25	–
M. smegmatis	–	17
C. albicans	–	–
Erwinia amylavora	15	20
Clavibacter michiganensis	15	20
K. pneumoniae 107210323	19	–
K. pneumoniae 53130623	20	–
K. pneumoniae 77270223	21	–
K. pneumoniae 60130323	22	–
K. pneumoniae 152310323	23	–
K. pneumoniae 12-13-14040423	24	–
K. pneumoniae 83180423	25	–
K. pneumoniae 152310323	23	–
K. pneumoniae 9030923	26	–
P. aeruginosa GIMC5015:PAKB6/2014	27	–
P. aeruginosa GIMC5016:PA1840/36/2015	28	–
P. aeruginosa GIMC5036:PA150608	29	–
MRSA 69181223_005	30	–

These results are consistent with the literature data [2-4].

Figure S3. HPLC chromatogram showing the purification of albomycin δ2. Chromatographic conditions: Luna C18(2) column (250 × 4.6 mm, 5 µm); mobile phase—(A) water and (B) acetonitrile; gradient—5-20% B (0-3 min), 20-60% B (3-9 min), 60-95% B (9-12 min), 95% B (12-16 min); flow rate—1.0 mL/min; detection—290 nm. The peak corresponding to albomycin δ2 eluted at 11.320 min.

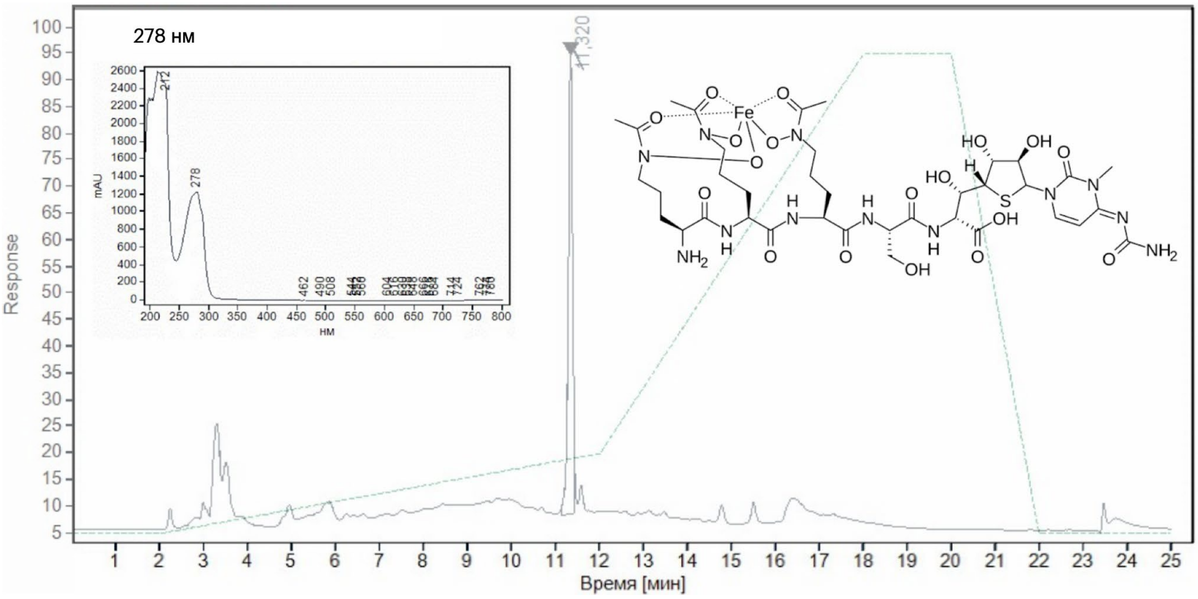

Figure S4. HPLC analysis of the deferoxamine E-containing fraction. Separation was achieved on a Luna C18(2) column (250 × 4.6 mm, 5 µm) using gradient elution with mobile phase A (water) and B (acetonitrile) at a flow rate of 1.0 mL/min. The gradient program was 5-20% B (0-3 min), 20-60% B (3-9 min), 60-95% B (9-12 min), and 95% B (12-16 min). Detection was performed at 272 nm. The peak corresponding to deferoxamine E eluted at 14.104 min.

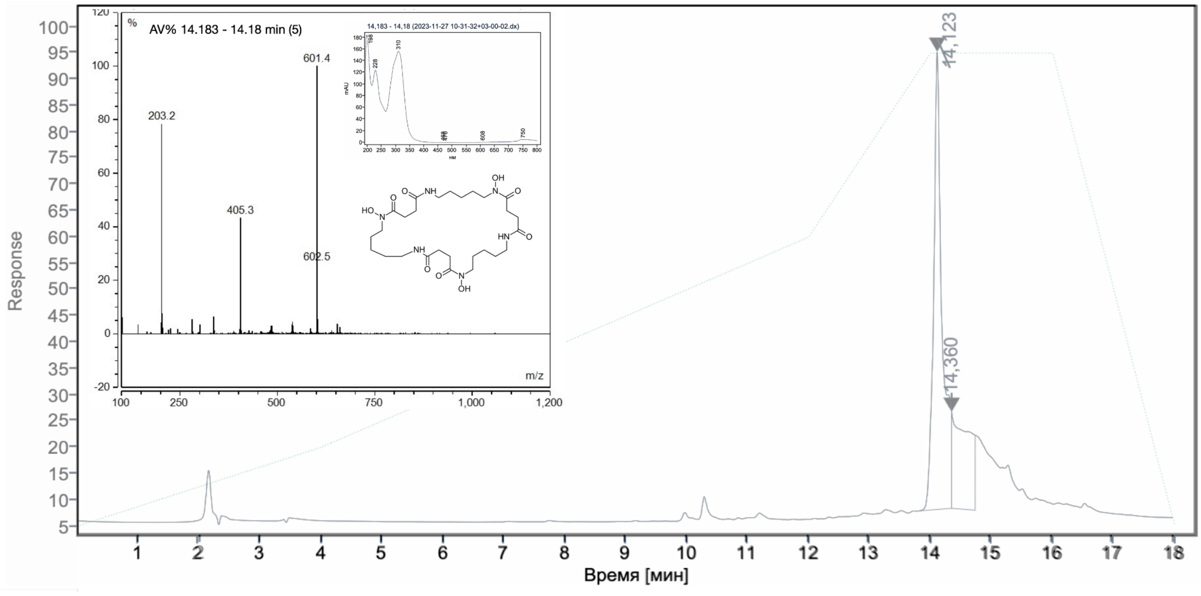

Figure S5. Detection of iron-chelating activity of deferoxamine E using the Chrome Azurol S (CAS) assay. The formation of a distinct orange halo around the sample well confirms the presence of a siderophore capable of removing iron from the blue CAS-Fe(III) complex.

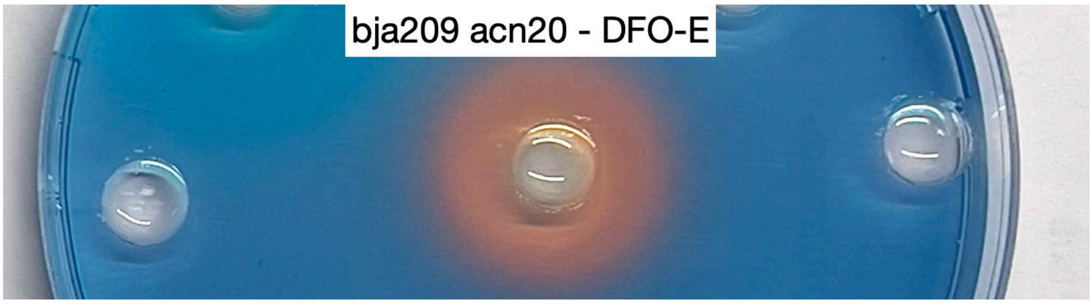

Figure S6. Antibacterial activity of desferrioxamine E against antibiotic-resistant *E. coli* strains. The assay compares the efficacy of desferrioxamine E against the following: Fluoroquinolone-resistant strains—*E. coli* Δ (quinolone-resistant mutant) and *E. coli* 5' (specific resistance profile); Nybomycin-resistant strain—*E. coli* a119e. *Streptomyces iakyrus* Pe6 (nybomycin-producing strain, reference [5]) was included as a control. The results suggest a potential mechanism of action distinct from conventional quinolones, possibly involving gyrase inhibition similar to nybomycin.

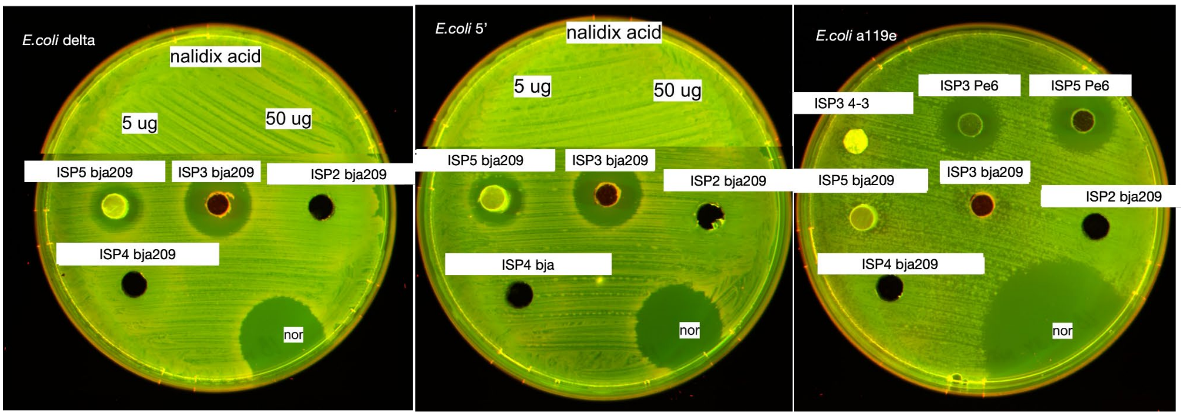

Table S5. Composition of modified media used to identify nutritional inducers of deferoxamine E and albomycin production in *S. globisporus* bja209. Media components (g/L) are listed for each variant (B0-B7). Key components tested include soluble starch (carbon source), KNO_3 (nitrogen), $MgSO_4$ (magnesium), iron citrate (iron), peptone (organic nitrogen), hydrolyzed casein (amino acids), and K_2HPO_4 (phosphate).

	B0	B1	B2	B3	B4	B5	B6	B7
Soluble starch	20		20	20	20	20	20	20
KNO3	1	1		1	1	1	1	1
MgSO4	0.5	0.5	0.5		0.5	0.5	0.5	0.5
Iron citrate	0.5	0.5	0.5	0.5		0.5	0.5	0.5
Peptone	20	20	20	20	20		20	
Hydrolyzed casein	5	5	5	5	5	5		
K2HPO4	0.5	0.5	0.5	0.5	0.5	0.5	0.5	0.5

Figure S7. Activity of crude broth of bja209 against *E. coli* JW5503 Δ*tolC* and *E. coli* BW25113 *lptD*. The agar plates were spotted with erythromycin, 5 µg/mL (Ery), and norfloxacin, 1 µg/mL (Nor). Katushka2S and TurboRFP signals visualized via ChemiDoc MP with red and green colors, respectively.

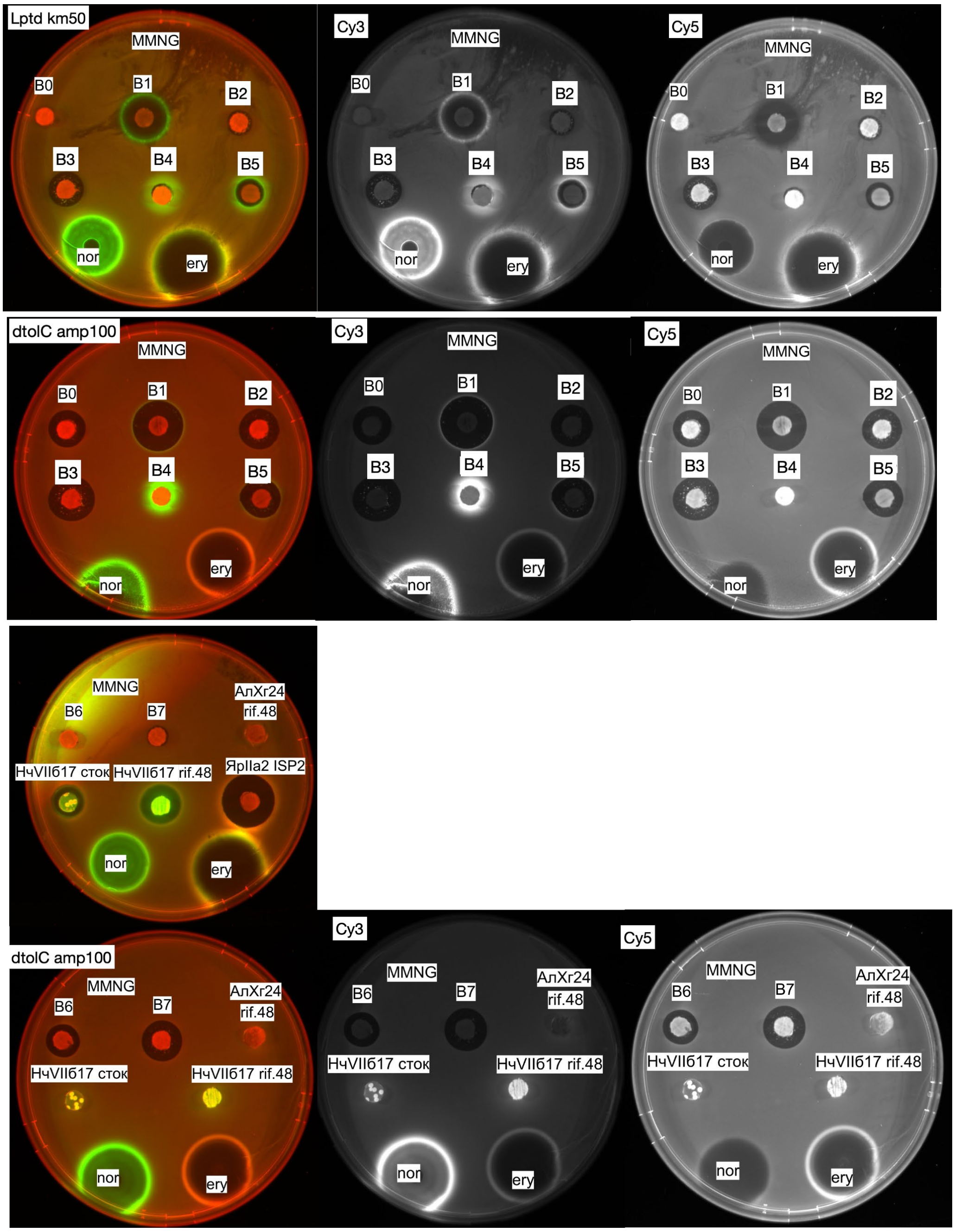

Figure S8. Analysis of gene expression in *S. globisporus* strains bja209 and 4-3 under iron-dependent conditions by RT-PCR. Electrophoretic separation of amplicons confirms iron-mediated repression of deferoxamine E biosynthesis genes (e.g., *desA*). Lanes: 1—DNA ladder; 2-7—bja209 cultured in iron-supplemented (B1) and iron-limited media (B4); 8-16—strain 4-3 under identical conditions. The *ropB* gene serves as a constitutive control.

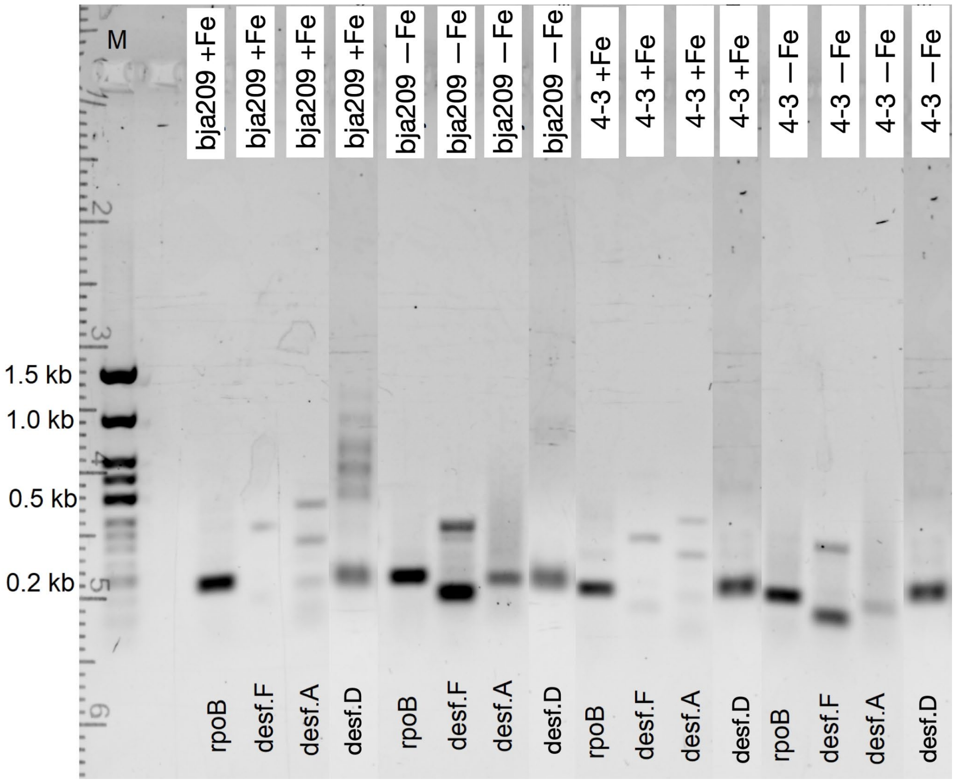

Supplementary References

1. Mota, L.J.; Tavares, P.; Sá-Nogueira, I. Mode of action of AraR, the key regulator of L-arabinose metabolism in Bacillus subtilis. *Mol. Microbiol.* **1999**, *33*, 476–489.
2. Volynkina, I.A.; Zakalyukina, Y.V.; Alferova, V.A.; Belik, A.R.; Yagoda, D.K.; Nikandrova, A.A.; Osterman, I.A. Mechanism-based approach to new antibiotic producer screening among actinomycetes in the course of the citizen science project. *Antibiotics* **2022**, *11*, 1198.
3. Pramanik, A.; Stroeher, U.H.; Krejci, J.; Standish, A.J.; Bohn, E.; Paton, J.C.; Braun, V. Albomycin is an effective antibiotic, as exemplified with Yersinia enterocolitica and Streptococcus pneumoniae. *Int. J. Med. Microbiol.* **2007**, *297*, 459–469.
4. Choi, O.; Cho, J.; Kang, B.; Lee, Y.; Kim, J. Negatively regulated aerobactin and desferrioxamine E by Fur in Pantoea ananatis are required for full siderophore production and antibacterial activity, but not for virulence. *Appl. Environ. Microbiol.* **2022**, *88*, 02405–02421.
5. Zakalyukina, Y.V.; Birykov, M.V.; Lukianov, D.A.; Shiriaev, D.I.; Komarova, E.S.; Skvortsov, D.A.; Osterman, I.A. Nybomycin-producing Streptomyces isolated from carpenter ant Camponotus vagus. *Biochimie* **2019**, *160*, 93–99.
